# Information gain modulates brain activity evoked by reading

**DOI:** 10.1038/s41598-020-63828-5

**Published:** 2020-05-06

**Authors:** Lauri Kangassalo, Michiel Spapé, Niklas Ravaja, Tuukka Ruotsalo

**Affiliations:** 1 Department of Computer Science, University of Helsinki, Helsinki, Finland; 2 Department of Psychology and Logopedics, University of Helsinki, Helsinki, Finland

**Keywords:** Language, Computer science

## Abstract

The human brain processes language to optimise efficient communication. Studies have shown extensive evidence that the brain’s response to language is affected both by lower-level features, such as word-length and frequency, and syntactic and semantic violations within sentences. However, our understanding on cognitive processes at discourse level remains limited: How does the relationship between words and the wider topic one is reading about affect language processing? We propose an information theoretic model to explain cognitive resourcing. In a study in which participants read sentences from Wikipedia entries, we show information gain, an information theoretic measure that quantifies the specificity of a word given its topic context, modulates word-synchronised brain activity in the EEG. Words with high information gain amplified a slow positive shift in the event related potential. To show that the effect persists for individual and unseen brain responses, we furthermore show that a classifier trained on EEG data can successfully predict information gain from previously unseen EEG. The findings suggest that biological information processing seeks to maximise performance subject to constraints on information capacity.

## Introduction

Human cognition and language are intimately related. On the one hand, the human cognitive system is sensitive to properties of the language input. Word frequency, recognition, ambiguity, and ease of processing drive attention, understanding, and memory^1–3^. On the other hand, human cognition has likely influenced the structure and properties of language itself^4^. For example, the large amount of redundancy in the structure of language facilitates learning^5^, while word lengths are optimised for efficient communication^6,7^.

Consequently, the demands on our cognitive system vary widely while reading, requiring us to dynamically allocate cognitive resources to process words and their syntactic and semantic dependencies. Eye-tracking studies show that while reading about the topic “cats” in a sentence such as “The cat is a carnivorous, usually furry mammal”, we will focus longer on informative, specific words like “cat”, “carnivorous”, and “mammal”, and less on uninformative words like “the”, “is”, “usually”, and “a”^8,9^. In order to efficiently gain topical meaning, we need to allocate our cognitive resources strategically towards those words that carry specific information.

EEG research has provided information about the dynamics of this strategic process by investigating brain responses to psycholinguistic anomalies. For example, a semantically unexpected closure in a sentence such as ‘He spread the warm bread with socks’ evokes a brain potential with a typical negative polarity and temporal signature - ca. 400 ms after viewing the last word^10^. This pattern of unexpected words evoking N400s has since been related to a process of semantic integration, with words failing to easily integrate consistently provoking N400s^11^. Early research on neurolinguistics suggested a dissociation between N400s and the P600, with syntactic anomalies, such as in a sentence like ‘The spoilt child throw the ball aside’, evoking a positive potential occurring ca. 200 ms after the N400^12^. However, further research cast doubt on the clear semantic/syntactic distinction, as it was shown that P600s could be provoked by processing difficulty and discourse complexity even without syntactic errors^13^.

While systematically introducing linguistic anomalies in controlled experiments has provided rich evidence on the temporal dynamics of sentence comprehension, the neurodynamics of natural reading at the discourse level remains little understood. Most experiments that have aimed towards natural language stimuli presented participants with words or sentences with little or no context; at best using individual sentences, words and their immediate context, such as words in a sentence around the stimuli word^14^. These studies have revealed N400 effects for word surprisal in a word sequence^15^ as well as amplified event-related potentials (ERPs) in response to general lexical statistics, such as frequency and word length^16,17^. Early research^18^ presented paragraphs of instructive text to participants, either with title, or without a title, the latter causing strong difficulty in comprehension. Analyzing the averaged ERPs to words from the latter condition, it was shown that sentences that were difficult to understand amplified the N400 to words within those sentences.

As a result, it is known that the statistical properties underlying language, cognition, and the event related potential to words are related, but the exact nature of this relationship remains elusive. While specific linguistic properties, such as word frequency, word length, position, and surprisal have been found to correlate with ERP components, these alone cannot sufficiently determine the synchronization and desynchronization of brain evoked activity as occurring at a discourse level. For example, while “carnivorous” is not only a lengthy adjective, it is also strongly specific to far fewer subjects than, for example, “usually”: it carries particular information regarding the topical context.

Here, we study whether the efficient coding principle^19,20^ can be used to explain linguistic processing in the human brain. According to the efficient coding principle, a cognitive information processing system aims to minimize the cost of perceptual error in order to detect generalizable and discriminative features in perceived stimuli when the system’s information processing capacities are limited^21,22^.

Previous studies have used efficient coding to explain the discrimination of low-level cognitive responses, such as visual or auditory perception^20,23^. We investigate whether the efficient coding principle can be used to explain higher-level discrimination tasks when reading natural language. In natural language, words can be understood as the units of a message and they code information in documents to convey (or signify) a more complex message. We hypothesize that in processing language the human cognitive system is continually engaged in discriminating between complex messages by allocating cognitive resources to the words that provide maximal information about the intended message.

We build on information theory and operationalize the discriminatory power of words by measuring the information gain of each word within a document corpora^20,24,25^. Information gain quantifies the reduction of uncertainty of the message (in this case, document) given a perceived message unit (word) from that document^26^. Intuitively, information gain answers the question “given a limited amount of message units (words), which units should be selected from a document to describe the information in that document most efficiently”. The full formal definition of the information gain model along with examples is provided in the supplementary material S1 and summarised in materials and methods under *information gain* subsection.

To study the effect of efficient coding of language on brain activity, an experiment connecting natural reading and brain activity was conducted. Information gain was computed for words in document context by using the entire English Wikipedia corpora and the EEG of participants was recorded while they were reading a sample of these documents. We hypothesised that the computed information gain of words modulates changes in ERPs indicating cognitive resource allocation following the efficient coding principle.

Specific ERP components have previously been related to specific stages of processing, from orthographic and lexical (P200, P300^27^), to semantic and syntactic levels (N400, P600^11,28–30^). If information gain would modulate the later (N400, P600) components, it could theoretically be due to the present word’s semantic content, and thus have bottom-up effects on information processing. On the other hand, if information gain modulated early (P200, P300) components occurring prior to semantic processing, current psycholinguistic theory suggests it must be the result of top-down effects of the preceding linguistic context. Efficient coding would predict that information gain should have top-down effects on information processing, as early detection of high information gain would enable divestment of cognitive resources from late semantic and syntactic linguistic stages. Thus, by exploring the specific linguistic stage during which information gain would affect the ERP, we expected to uncover important information regarding the degree to which information gain modulated bottom-up or top-down word processing.

In our analysis we first focus on whether information gain modulates the brain activity evoked by reading a word. We then investigate whether the specific components are reliably modulated, after controlling for previously known linguistic properties. This may uncover whether information gain can be temporarily dissociated between components, and furthermore may hint towards the top-down and bottom-up effects of information gain. We then investigate whether not only are ERPs modulated by information gain, they can also predict by reverse inference the information gain from a previously unseen sample of EEG data in a single-trial machine learning setting.

## Results

**Figure 1:**
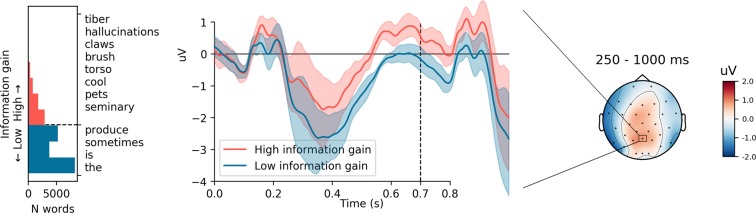
Left: Occurrences of information gains for all words presented to the participants, with the dashed line marking the split between words associated with high and low information gain. On the right side of the plot are displayed example words ordered by their information gain (descending). Centre: Grand average event-related potential at the Pz channel for words associated with high/low information gain. The shaded areas represent a 95% confidence interval. The dashed line marks the onsets of the next stimulus. Right: A scalp topography of the differences of ERPs between low information gain words and high information gain words for the time interval 250–1000 ms post-stimuli. Each contour line marks a 1 *μV* difference in voltage. The Pz electrode is highlighted with a rectangle.

Visual inspection of the grand average ERPs suggested two distinct states in which low and high information gain modulated the ERP. As shown in Movie S1 (an animated scalp topography of the difference wave), low information gain evoked increased frontal positivity at ca. 200 ms, which we identified with a P200, maximal over Fz. Following ca. 250 ms, this was replaced by a long lasting (until ca. 700 ms) positivity over parietal sites (see Fig. 1 [right panel]). As illustrated in Fig. 1 (middle panel), this could be related to a unitary, long-lasting Early Positive Shift (EPS) affecting the ERP over parietal electrodes from ca. 250 ms.

### The effect of information gain on the early positive shift

To identify whether information gain accounts for this EPS, a linear mixed model was defined, with word log-frequency (given the whole corpus), word length (number of characters), word class (functional/content word), and document preference (subjectively selected interest of a participant towards a topic of the document) as factors. Information gain was added as a fixed effect, and the increase in model fit was calculated using the *χ*^2^. As summarised in Table 1, information gain significantly contributed to model fit for the EPS, with *χ*^2^ = 5.98, p < 0.05. In addition to information gain, the EPS was substantially affected by Word length, *β* = 0.064, and Word log-frequency, *β* = 0.072 but not Word class or Document preference, *β*s < 0.012.

**Table 1:** Results of statistical tests for the Early Positive Shift (EPS).

Effect	Effect slope	*χ*^2^
Word length	0.064	
Word log-frequency	0.072	
Word class	0.011	
Document preference	0.010	
Information gain	0.101	5.98*
Marginal *R*^2^/Conditional *R*^2^	0.009/0.028	

Columns from left to right: the name of the tested fixed effect; the standardised estimated slope for the effect; and the *χ*^2^ statistic representing the difference in deviance between a model which includes information gain as an effect and a model which does not. Significance is coded as with a star:’*’. The performance of the model is displayed as Marginal (variance explained by fixed effects only) and Conditional (variance explained by fixed and random effects) *R*^2^ values.

Alternatively, the seemingly long-lasting EPS could be the result of a summation of components that separately contribute to the common channel: i.e., an increased P300, a decreased N400, and an increased P600. For this reason, analysis for effects of separate ERP components were conducted.

### 0 effect of information gain on ERP-components

Four additional linear mixed models were defined for the ERP-components P200, P300, N400, and P600. The models were formulated and the significance was tested as with the EPS.

As summarised in Table 2, information gain significantly contributed to model fit for N400, *χ*^2^ = 7.73, p < 0.05, but not the other components. Two of the controlled factors, Word class and Document preference, did not contribute substantially to any of the components, *β*s < 0.020. Word length, in contrast, affected all components, from the P200, *β* = 0.030, to more than doubling in effect on N400, *β* = 0.082, before decreasing for the P600, *β* = 0.030. Word log-frequency had a substantial effect on the N400, *β* = 0.100, and a smaller effect on P200, *β* = 0.062, and P600, *β* = 0.040.

**Table 2:** Results of statistical tests for each of the studied ERP-components.

Component	Effect	Effect slope	*χ*^2^
P200	Word length	0.030	
	Word log-frequency	0.062	
	Word class	0.018	
	Document preference	−0.006	
	Information gain	0.074	4.68
m *R*^2^/c *R*^2^		0.003/0.016	
P300	Word length	0.068	
	Word log-frequency	0.025	
	Word class	−0.009	
	Document preference	0.011	
	Information gain	0.053	2.72
m *R*^2^/c *R*^2^		0.007/0.079	
N400	Word length	0.082	
	Word log-frequency	0.100	
	Word class	0.009	
	Document preference	0.013	
	Information gain	0.116	7.73*
m *R*^2^/c *R*^2^		0.010/0.035	
P600	Word length	0.030	
	Word log-frequency	0.040	
	Word class	0.019	
	Document preference	0.004	
	Information gain	0.076	3.46
m *R*^2^/c *R*^2^		0.006/0.012	

Columns and notation as in 1. The p-values have been Bonferroni corrected with *m* = 4.

The effect of information gain remained positive and high on all of the studied components. It varied between *β* = 0.053 (P300) and *β* = 0.116 (N400). We found a significant effect for the N400 component, *χ*^2^ = 7.73, p < 0.05. The effect of information gain was non-significant for the P200, *χ*^2^ = 4.68, p = 0.12.

Thus, even controlling for known effects of word-length, word log-frequency, and word class, information gain was shown to provide additional explanatory value in the EPS and the N400 component.

### Comparison of information gain with other statistical measures

Many of the statistical measures used for studying language in a neurophysiological context were found to highly correlate with each other. Table 3 displays the correlations between information gain, log-frequency, and word length. A substantial correlation is found with all of the measures, with information gain and log-frequency being highly inversely correlated, with a Pearson correlation −0.98. Due to this intercorrelatedness, it is likely that no single effect can explain all of these differences, and the differences thus reflect a mixture of multiple simultaneous neural processes affected by different characteristics of words.

**Table 3:** Pearson correlations between linguistic measures.

	Word length	Word log-frequency	Information gain
Word length	1.0	−0.72	0.7
Word log-frequency	−0.72	1.0	−0.98
Information gain	0.7	−0.98	1.0

**Figure 2:**
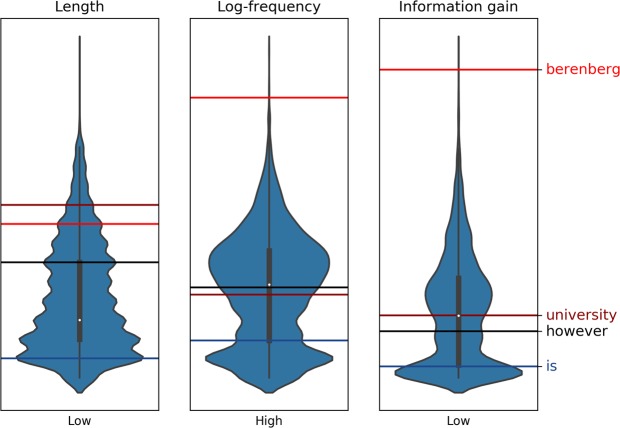
Comparison of linguistic measures for words displayed to the participants in the study. Distributions for length, log-frequency and information gain are depicted as violin plots. The words *berenberg*, *university*, *however*, and *is* are marked by color-coded horizontal lines in each distribution. Note that the vertical axis of log-frequency has been reversed for easier comparison of the distributions. The jaggedness of the length distribution is caused by the distribution being discrete, while the other two are continuous.

To gain insight to the interactions between the three measures, the shape of their distributions and rank of words given by each measure were compared. Figure 2 depicts the distributions of the measures, with the locations of three words *berenberg*, *university*, *however*, and *is* plotted as color-coded horizontal bars in each plot. These words were chosen to depict the differences of each measure with relation to information gain. The word *berenberg* has the highest information gain, with the others having a lower information gain, in a descending order.

It can be clearly observed that log-frequency and information gain behave similarly, while the distribution of word lengths somewhat differs from these two measures. Information gain tends to push words with extreme values away from the distribution mean (e.g. *berenberg* to the top, *is* to the bottom) as compared to log frequency. Also, *university* and *however* have switched ranks, with *university* ranking higher than *however*, while the opposite is true for log-frequency. This is explained by information gain taking in to account the document context in which the words appear. The word *however* may be rarer in the full corpus, but is not as good at discriminating documents as *university*. Despite the high correlation between the measures, our aim was to determine the independent effect of information gain. Therefore, the effects of word length and log-frequency were controlled when testing the significance of information gain.

### Predicting information gain from ERPs

**Figure 3:**
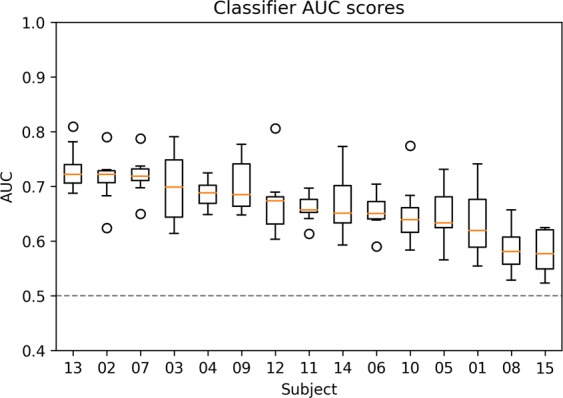
The classifiers’ AUC scores for each subject and reading task. The dashed line marks the performance of a random-permutation classifier.

The EPS was found to be parsimonious and the effect was present across ERP components. In order to study whether the EPS could be used as a neural marker for information gain, we built a predictive model to reveal the effectiveness of the EPS in estimating information gain in a single-trial classification setting. Per-subject linear classifiers which predicted the information gain class (low/high) of ERPs were trained. A single-trial prediction setting was used in which ERPs used for validating the classifier were not used in training the classifier (The specific evaluation setting is given in materials and methods under *prediction* subsection). The average classifier AUC score over all participants was 0.641. The classifiers of all subjects performed significantly better than random-permutation classifiers (AUC = 0.5, p < 0.01). The AUC scores of the classifiers can be seen in Fig. 3.

Illustrating classification results, Table 4 shows examples of words from three randomly selected documents, with the words grouped by their predicted information gain. The prediction confidence for words to belong to the low information gain class was computed over all participants and reading tasks. The first two columns show the five words which had the highest classifier confidence for belonging to the low information gain class and the five words with the lowest actual information gain (ground truth) in the selected documents. The remaining two columns show the five words which had the highest classifier confidence for belonging to the high information gain class and highest actual information gain. For example, the word ’housecat’ had the second highest classifier confidence for belonging to the low information gain class of all the words in the document about cats, and the same word had the lowest information gain of the words in the document.

**Table 4:** Top/bottom 5 words per topic sorted by classifier confidence (predicted) for class membership (low information gain/high information gain) and by actual information gain i.e. ground truth (true).

Document	High information gain	High Information gain	Low information gain	Low information gain
	Predicted	True	Predicted	True
Volcano	eruption	troposphere	to	the
	temperature	droplets	can	and
	tectonic	magma	is	in
	surface	plumes	lower	a
	atmosphere	crust	on	of
Cat	killing	housecat	with	the
	housecat	felids	for	and
	mammal	purring	as	in
	indoor	mewing	such	a
	despite	felines	being	of
Rome	michelangelo	bramante	to	the
	bramante	bernini	chapel	and
	province	sistine	for	in
	baroque	tiber	in	a
	architecture	michelangelo	was	of

A clear difference between the words in the high/low information gain predicted classes was observed. The words in the low predicted information gain column are informative to the source document (e.g.’eruption’,’tectonic’,’atmosphere’ for document’volcano’) and resemble the ground truth. On the other hand, words in the high predicted information gain class tend to be short functional words, which are intuitively not very informative. The full list of top 5 predicted/ground truth words for all documents and classes can be found in Table S3.

## Discussion

We set out to study whether information gain, a measure based on the efficient coding theory^19,20^, could explain brain activity occurring during natural reading. Our findings show that computationally quantified information gain modulates brain activity evoked by reading natural language, suggesting that the efficient coding principle may partially explain high-level language processing. We furthermore show that it is possible to predict information gain from individual event-related potentials with machine learning methods.

Since words with high information gain are frequent in only a few documents and low information gain words are infrequent or non-existing in most of the documents in a corpus, information gain can be seen as a measure of the context specificity of a word. For instance, the high information gain words for the document Rome  as displayed in Table 4 are *bramante*, *bernini*, *sistine*, *tiber*, and *michelangelo*. At a first glance it might seem odd that the word *rome* is not one of the five words with the highest information gain in the document. While the word *rome* is undoubtedly relevant to the Rome document, it is not the most context specific word of the document. For instance, the word *rome* may appear in documents discussing popular travel destinations, football teams, or religion. The high information gain words, on the other hand, are tightly connected to the topic of the document; the capital of Italy and an ancient empire. Thus, *bramante*, *bernini*, *sistine*, *tiber*, and *michelangelo* are more specific and have a high information gain to discriminate the underlying message that the document conveys.

Analysis of the ERPs showed low information gain to evoke an Early Positive Shift (EPS), affecting the potential in the P200-P600 range. Separate analysis of the P200, P300, N400, and P600 while controlling for lower-level language properties, showed information gain to contribute particularly to the N400-component. As, however, the effect of information gain was similar across P300, N400, and P600 components, an explanation involving a single information gain-evoked EPS describes the effect more parsimoniously than one involving independently contributing effects.

To further establish the validity of ERPs predicting information gain, we used a classifier to predict which words had high and low information gain from single subject, single epochs of EEG data. Given the degree of noise inherent in EEG data, we showed surprisingly strong performance for the predictions. Thus, classifiers were able to capture the difference in ERPs related to low and high information gain.

These findings show a striking similarity between computational models of information gain and the EPS suggesting that human information processing is corresponding to statistically quantifiable informativeness of words^20,31^. Interestingly, a similar mechanism has been shown to be effective in various artificial information processing systems, such as search engines, automatic classification systems, and natural language processing methods, for discriminating and estimating informativeness of words in their document context^32–34^.

In terms of cognitive neuroscience, we show that brain activity synchronised with word presentation is modulated by the statistical properties that determine the informativeness of language. While consistent with early experimental findings from perception^35^ and neurolinguistics^36^, here we show the process occurring in natural language reading, as opposed to artificially constructed sentences. Furthermore, we provide evidence that even a single second of EEG data contains all required information to predict information gain. Not only is this a powerful indication that inverse inference^37^ is feasible, it also suggests many possibilities for applications that would profit from non-intrusive online extraction of perceived text informativeness.

Interestingly, the ERP analysis suggests an early, top-down effect of information gain that influences word reading well before semantic processing. Given that semantic repetition is known to affect the ERP at a much later stage than the observed EPS^38^, this indicates information gain likely has a top-down effect. In other words, the statistical structure of language provides a pre-existing context due to which high or low information gain is *expected* to occur.

While natural reading provides a richer, more ecological setting for the study of language comprehension than classic neurolinguistic designs, it does require critical review of various confounds that could potentially provide alternative accounts to our results. Various studies have, for example, pointed out that low level language properties such as word length and word frequency^39^ modulate ERPs. The seriousness of this is compounded by the fact that words that are usable in many contexts (i.e. have low information gain) tend to be frequently used and short^7^. On the other hand, one could argue that both word length and word frequency are manifestations of the statistical attribute of information gain, rather than the other way around, and therefore that information gain could provide a more parsimonious explanation of the previously observed effects. However, to rule out that such lower-level attributes provide alternative accounts, we statistically controlled for them. This showed that although word frequency and length were associated with the observed voltage fluctuations in the ERPs, information gain had a unique contribution to the measured ERPs, particularly as an early positive shift starting at circa 250 ms post-stimuli.

Our experimental design involved presentation of interwoven sentences from a preferred and a non-preferred document. The reasoning behind this design decision was that we aimed to control for preferential processing. For example, a strong, confounding effect of order could easily affect ERPs if participants knew in advance that information appearing would be irrelevant, causing them to disengage from the task. The results suggest that we were successful in this objective as document preference had no effect on the results (see Table 2). However, to further investigate a potential effect of document preference on semantic integration, further experiments could contrast natural sentences with sentences in scrabled order to reveal effects of sentence structure. Conversely, an experiment with deep priming for a particular topic by presenting a pre-reading task could reveal the effect of additional information gain against a predetermined baseline.

In fact, several higher-order linguistic properties have been shown to affect ERPs in language comprehension. Surprisal, predictability, and expectancy of language have been shown to affect the N400 when presented in a sentence context and controlled for semantic anomalies^18,40,41^. However, our results show that information gain, when observed at discourse level using natural language from documents as stimuli, is associated with consistent, but different, positive shift (EPS).

Information gain in general also provides theoretically motivated and sound computational measure that can mathematically account for a diverse range of information processing phenomena, from how we prioritise low-information gain visual features over those that are redundant^42^ to how motivation is provoked by it^43^. While this type of information-processing explanation has gradually lost prominence in the age of neuroimaging, the EPS constitutes the neurological correlate of information gain of words. This effect appears consistent, but the precise nature and localization of this effect remains to be determined.

In conclusion, our results suggest that the biological correlates to language processing follow the efficient encoding principle. Changes in synchronization and desynchronization underlying event related potentials reflect a maximization process towards informative, context specific words.

Appendix

## Materials and Methods

### Measures

#### Information gain

Formally, the information gain of the word *w* the difference between a priori entropy over a document collection and entropy over the documents conditioned with *w*: $IG(D|w)=H(D)-H(D|w)$. The entropies are computed with generative document probabilities obtained with the query likelihood model and unigram language models^26^. The full formal definition of information gain can be found in the supplementary material S1.

#### Other measures

Other measures used in the statistical tests were word length (number of characters), word log-frequency (in the whole corpus), word class (functional/content word), and document preference. Document preference was a binary digit indicating a participant’s interest towards a topic, as designated by the participant during the neurophysiological experiment.

### Neurophysiological experiment

#### Participants

Seventeen participants were recruited to participate in the experiment. All were adults, and most were postgraduate students from University of Helsinki and Aalto University. Their participation was contingent on showing sufficient fluency in English, which was tested prior to the experiment via the Cambridge English “Test your English–Adult Learners” test (M = 23.53, SD = 1.23, out of 25 maximum (https://www.cambridgeenglish.org/in/test-your-english/adult-learners/). Participants received full instruction on the nature of the study, and their rights as participants, including the right to withdraw without fear of any negative consequences, in line with the Declaration of Helsinki. They all signed informed consent prior to the study and received two movie tickets as compensation after the study. Data from two participants were removed due to technical issues occurring during the experiment. Of the remaining participants, eight were female and seven were male.

#### Stimuli and procedure

Participants were asked to read 16 random documents drawn from a pool of 30 (see Table S3). They each completed eight reading tasks, and each task consisted of a pair of two documents. Tasks started by displaying the topics of the two documents and requesting participants to indicate which of the two topics they found the most interesting. They were then instructed to keep their answer in mind while reading everything that was displayed during the next trials. Trials involved, for each pair of documents, the set of sentences being presented in alternating document order. Thus, the first trial involved sequential presentation of each word in the first sentence of the first document, followed by the first sentence of the second document, while in the second trial, the second sentence of the second document was read before the second sentence of the first document. At the end of each pair of sentences, two validations were provided to ascertain participants 1) read the sentences and 2) remembered the topic that was to be kept in mind. For the former, they were shown one of the sentences (randomised), with one of the nouns or verbs missing and replaced by question marks, and asked to fill in the missing word. For the latter, they were asked to recall the topic to be kept in mind by typing it. Feedback regarding their performance on these two tasks was shown in order to facilitate performance. Following an inter-trial interval of 1 s, the next pair of sentences was shown. This procedure was repeated for the first six sentences of both of the documents in the reading task.

Within each trial, the pair of sentences was shown using a rapid serial visual presentation paradigm, involving individual words (punctuation words were not shown) sequentially shown at a rate of 1 word/700 ms (SD = 0.3 ms). These were shown against a black, rectangular pattern mask, designed to minimise differences in luminance as a function of word length. Different, letter-like pattern masks were shown before the beginning of the first word and after the final word for each sentence to indicate separations between trials and sentences. The entire experiment, including instruction, psychophysiological preparation and three self-timed breaks took approximately an hour and forty minutes.

The study was fully designed and performed in accordance with the relevant guidelines and regulations, particularly those set out in the Declaration of Helsinki pertaining to the ethical treatment of human subjects. Participants were fully briefed as to the nature and purpose of the study prior to the experiment, signed informed consents, and were instructed on their rights as participants, including the right to withdraw from the experiment at any time without fear of negative consequences. All experimental protocols were approved by the University of Helsinki Ethical Review Board in the Humanities and Behavioural Sciences.

### Data preprocessing and analysis

#### EEG recording and preprocessing

EEG data were recorded from 32 Ag/AgCl electrodes mounted on 32 equidistant positions of an elastic cap (EasyCap), amplified and digitised using a BrainProducts QuickAmp USB 32. Continuous EEG recordings were band-pass filtered between 0.25–35 Hz using a Firwin1 filter. Data were then epoched with 200 ms of baseline activity before and 1000 ms data following word onset, with the average over the baseline subtracted from each epoch. A custom artifact rejection procedure was based on per-participant voltage thresholds based on centrally located channels (F3, Fz, F4, FC1, FC2, C3, Cz, C4, CP1, CP2, P3, Pz, and P4). This involved identification of 80th percentile of highest absolute maximum voltage between −200 and 700 ms, resulting in thresholds ranged from 25 to 67 *μV* between participants. Channels were marked as bad based on a similar procedure involving computation of the absolute maximum voltage per channel and trial. Channels producing invalid epoch rates over 20% were marked and interpolated from neighbouring channels. Following preprocessing, the average number of epochs per participant was 1550. Individual statistics of number of interpolated channels and numbers of dropped epochs may be found in Table S2.

#### Statistical analysis

The significance of the effect of information gain on ERPs was tested with likelihood ratio tests on Linear Mixed Models (LMMs). LMMs were chosen over the traditional ANOVA methods in order to consider the non-independencies introduced by the natural language text used as stimuli. Separate models were fitted for the EPS as well as each ERP component. The dependent variable in each of these models was the average voltage over each components’ time windows, defined as: EPS (250–700 ms), P200 (100–250 ms), P300 (250–350 ms), N400 (350–500 ms), and P600 (500–800 ms). The time intervals for the ERP-components were defined based on visual inspection of the ERPs and literature^10,29,30,44^. Measurements from the Fz channel were used to compute the averages for the P200 component, while measurements from Pz were used for all others.

To avoid common pitfalls resulting from Type I errors, the LMMs were designed using the “keep it maximal”-principle presented by Barr *et al*.^45^. Fixed effects in these models were information gain (continuous), word length, log word frequency in corpus, and document preference (as specified by the participant during the reading experiment, binary). Random effects included intercepts for subjects and stimuli (words). Word length and log frequency were included as effects because they have been shown to have an effect on word-related ERPs^39^. The models initially had the maximal randomâ€ effects structures permitted by the design, but to achieve convergence the random effects explaining the least variance were dropped until convergence was achieved^45,46^. The converged models were evaluated with likelihood ratio tests of the alternative hypotheses model with information gain as a fixed effect, and a null hypothesis model, which excluded the information gain effect, but was otherwise the same. The model formulations and the steps taken to achieve convergence are specified in supplementary material S1.

#### Prediction

Regularised Linear Discriminant Analysis (LDA) was utilised to learn linear classifiers to separate ERPs associated with words in the low and high information gain classes. LDA has been previously used successfully in single-trial ERP classification^47,48^. Binary LDA classifiers were trained for each participant separately due to the individual differences in EEG measures. The data used for classifier training consisted of a vector representation of the ERP and a binary label indicating whether the ERP was associated with a low or high information gain word. The vector representation of the ERP was composed of the data from all available electrodes, and time windows spanning the whole trial (0–1000 ms). Feature engineering is further specified in the supplementary material S1.

Classifier performance was evaluated by leave-one-out validation. The data was split to eight blocks coinciding with the reading tasks, of which seven were used for training the classifier and one for testing it. The performance of the classifier was measured with Area Under ROC Curve (AUC). This measure was chosen because AUC combines the true positive and false positive rate, and thus gives better estimates of the performance when the classes are imbalanced as in this case. To ensure that the classifiers were performing better than random, the AUC scores of the classifiers were compared with the AUCs of classifiers trained with randomly permuted class labels. With a sufficiently high number of permutations this produces permutation-based p-values^49^. We ran *k* = 1000 permutations for each subject, which leads to a minimum possible p-value of 0.001^50^. The details of the evaluation can be found in the Supplementary Material S1.

## Supplementary information

Supplementary information

Movie S1.

## Data Availability

The datasets generated during and/or analysed during the current study are available from the corresponding author on reasonable request.

## References

[1] Jurafsky, D. Probabilistic modeling in psycholinguistics: Linguistic comprehension and production. In *Probabilistic linguistics*, 39–96 (MIT Press (2003).

[2] Griffiths, T. L. Rethinking language: How probabilities shape the words we use. *Proceedings of the National Academy of Sciences* **108**, 3825–3826, https://www.pnas.org/content/108/10/3825, https://www.pnas.org/content/108/10/3825.full.pdf (2011). 10.1073/pnas.1100760108 PMC3053996 21368209

[3] Hulme C (1997). Word-frequency effects on short-term memory tasks: Evidence for a redintegration process in immediate serial recall. Journal of Experimental Psychology: Learning, Memory, and Cognition.

[4] Christiansen MH, Chater N (2008). Language as shaped by the brain. Behavioral and brain sciences.

[5] Nicolaci-da Costa A, Harris M (1983). Redundancy of syntactic information: An aid to young children’s comprehension of sentential number. British Journal of Psychology.

[6] Piantadosi, S. T., Tily, H. &Gibson, E. Word lengths are optimized for efficient communication. *Proceedings of the National Academy of Sciences* **108**, 3526–3529 http://www.pnas.org/lookup/doi/10.1073/pnas.1012551108 (2011). 10.1073/pnas.1012551108 PMC3048148 21278332

[7] Lewis ML, Frank MC (2016). The length of words reflects their conceptual complexity. Cognition.

[8] Rayner K (1998). Eye movements in reading and information processing: 20 years of research. Psychological bulletin.

[9] Rayner K, Duffy SA (1988). On-line comprehension processes and eye movements in reading. Reading research: Advances in theory and practice.

[10] Kutas M, Hillyard SA (1980). Reading senseless sentences: brain potentials reflect semantic incongruity. Science.

[11] Kutas, M. & Federmeier, K. D. Thirty Years and Counting: Finding Meaning in the N400 Component of the Event-Related Brain Potential (ERP). *Annual Review of Psychology* **62**, 621–647, https://www.annualreviews.org/doi/10.1146/annurev.psych.093008.131123 (2011). 10.1146/annurev.psych.093008.131123 PMC4052444 20809790

[12] Hagoort P, Brown C, Groothusen J (1993). The syntactic positive shift (sps) as an erp measure of syntactic processing. Language and cognitive processes.

[13] Kaan E, Swaab TY (2003). Repair, revision, and complexity in syntactic analysis: An electrophysiological differentiation. Journal of cognitive neuroscience.

[14] Brennan, J. Naturalistic sentence comprehension in the brain. *Language and Linguistics Compass* **10**, 299–313, https://onlinelibrary.wiley.com/doi/abs/10.1111/lnc3.12198, https://onlinelibrary.wiley.com/doi/pdf/10.1111/lnc3.12198 (2016).

[15] Frank SL, Otten LJ, Galli G, Vigliocco G (2015). The erp response to the amount of information conveyed by words in sentences. Brain and language.

[16] Hauk, O. & Pulvermuller, F. Effects of word length and frequency on the human event-related potential. *Clinical Neurophysiology* **115**, 1090–1103. http://www.sciencedirect.com/science/article/pii/S1388245703004759 (2004). 10.1016/j.clinph.2003.12.020 15066535

[17] Armeni, K., Willems, R. M. &Frank, S. L. Probabilistic language models in cognitive neuroscience: Promises and pitfalls. *Neuroscience & Biobehavioral Reviews* **83**, 579–588, http://www.sciencedirect.com/science/article/pii/S0149763416307898 (2017). 10.1016/j.neubiorev.2017.09.001 28887227

[18] George MS, Mannes S, Hoffinan JE (1994). Global semantic expectancy and language comprehension. Journal of cognitive neuroscience.

[19] Barlow, H. B. Possible principles underlying the transformation of sensory messages. *Sensory Communication* 217–234 (1961).

[20] Sims, C. R. Efficient coding explains the universal law of generalization in human perception. *Science* **360**, 652–656 http://science.sciencemag.org/content/360/6389/652 (2018). 10.1126/science.aaq1118 29748284

[21] Shepard RN (1987). Toward a universal law of generalization for psychological science. Science.

[22] Chater N, Vitányi PM (2003). The generalized universal law of generalization. Journal of Mathematical Psychology.

[23] Wei, X.-X. & Stocker, A. A. Lawful relation between perceptual bias and discriminability. *Proceedings of the National Academy of Sciences* **114**, 10244–10249, https://www.pnas.org/content/114/38/10244, https://www.pnas.org/content/114/38/10244.full.pdf (2017). 10.1073/pnas.1619153114 PMC5617240 28874578

[24] Shannon CE (1948). A mathematical theory of communication. Bell system technical journal.

[25] Wong SKM, Yao YY (1992). An information-theoretic measure of term specificity. Journal of the American Society for Information Science.

[26] Song, F. & Croft, W. B. A general language model for information retrieval. In *Proceedings of the eighth international conference on Information and knowledge management - CIKM* ’99, 316–321 (ACM Press, Kansas City, Missouri, United States, http://portal.acm.org/citation.cfm?doid=319950.320022 (1999).

[27] Holcomb PJ, Grainger J (2006). On the time course of visual word recognition: An event-related potential investigation using masked repetition priming. Journal of cognitive neuroscience.

[28] Evans, K. M. & Federmeier, K. D. The memory that’s right and the memory that’s left: Event-related potentials reveal hemispheric asymmetries in the encoding and retention of verbal information. *Neuropsychologia* 45, 1777–1790, http://www.sciencedirect.com/science/article/pii/S0028393207000073 (2007). 10.1016/j.neuropsychologia.2006.12.014 PMC2758159 17291547

[29] Sutton, S., Braren, M., Zubin, J. & John, E. R. Evoked-Potential Correlates of Stimulus Uncertainty. *Science* **150**, 1187–1188, http://science.sciencemag.org/content/150/3700/1187 (1965). 10.1126/science.150.3700.1187 5852977

[30] Osterhout, L. & Holcomb, P. J. Event-related brain potentials elicited by syntactic anomaly. *Journal of Memory and Language* **31**, 785–806, http://linkinghub.elsevier.com/retrieve/pii/0749596x9290039Z (1992).

[31] Eugster, M. J. A. *et al*. Natural brain-information interfaces: Recommending information by relevance inferred from human brain signals. *Scientific Reports* **6**, 38580. https://www.nature.com/articles/srep38580 (2016). 10.1038/srep38580 PMC5143956 27929077

[32] Ponte, J. M. & Croft, W. B. A language modeling approach to information retrieval. In *Proceedings of the 21st Annual International ACM SIGIR Conference on Research and Development in Information Retrieval*, SIGIR ’98, 275–281 (ACM, New York, NY, USA, http://doi.acm.org/10.1145/290941.291008 (1998).

[33] Berger, A. L., Pietra, V. J. D. &Pietra, S. A. D. A maximum entropy approach to natural language processing. *Comput. Linguist*. **22**, 39–71, http://dl.acm.org/citation.cfm?id=234285.234289 (1996).

[34] Kangassalo, L., Spapé, M., Jacucci, G. & Ruotsalo, T. Why do users issue good queries? neural correlates of term specificity. In *Proceedings of the 42nd International ACM SIGIR Conference on Research and Development in Information Retrieval*, sigir **19**, 375–384 (Association for Computing Machinery, New York, NY, USA, 10.1145/3331184.3331243 (2019).

[35] Sutton S, Tueting P, Zubin J, John ER (1967). Information delivery and the sensory evoked potential. Science.

[36] Friedman, D., Simson, R., Ritter, W. & Rapin, I. The late positive component (P300) and information processing in sentences. *Electroencephalography and Clinical Neurophysiology* **38**, 255–262, https://www.clinph-journal.com/article/0013-4694(75)90246-1/abstract (1975). 10.1016/0013-4694(75)90246-1 46803

[37] Poldrack RA (2011). Inferring mental states from neuroimaging data: from reverse inference to large-scale decoding. Neuron.

[38] Eugster, M. J. *et al*. Predicting term-relevance from brain signals. In *Proceedings of the 37th international ACM SIGIR conference on Research & development in information retrieval*, 425–434 (ACM (2014).

[39] Hauk, O. & Pulvermuller, F. Effects of word length and frequency on the human event-related potential. *Clinical Neurophysiology* **115**, 1090–1103, http://www.sciencedirect.com/science/article/pii/S1388245703004759 (2004). 10.1016/j.clinph.2003.12.020 15066535

[40] Davenport T, Coulson S (2011). Predictability and novelty in literal language comprehension: an erp study. Brain research.

[41] Frank SL, Otten LJ, Galli G, Vigliocco G (2013). Word surprisal predicts n400 amplitude during reading. In Proceedings of the 51st Annual Meeting of the Association for Computational Linguistics.

[42] Attneave F (1954). Some informational aspects of visual perception. Psychological review.

[43] Berlyne DE (1957). Conflict and information-theory variables as determinants of human perceptual curiosity. Journal of experimental psychology.

[44] Hagoort, P. & Kutas, M. Electrophysiological insights into language deficits. In *Handbook of neuropsychology*, vol. 10, 105–134 (Elsevier (1995).

[45] Barr, D. J., Levy, R., Scheepers, C. & Tily, H. J. Random effects structure for confirmatory hypothesis testing: Keep it maximal. *Journal of Memory and Language* **68**, 255–278, http://www.sciencedirect.com/science/article/pii/S0749596X12001180 (2013). 10.1016/j.jml.2012.11.001 PMC3881361 24403724

[46] Matuschek, H., Kliegl, R., Vasishth, S., Baayen, H. &Bates, D. Balancing type i error and power in linear mixed models. *Journal of Memory and Language* **94**, 305–315, http://www.sciencedirect.com/science/article/pii/S0749596X17300013 (2017).

[47] Muller, K.-R. et al. Machine learning for real-time single-trial EEG-analysis: From brainâ€“computer interfacing to mental state monitoring. *Journal of Neuroscience Methods* **167**, 82–90, http://www.sciencedirect.com/science/article/pii/S0165027007004657 (2008). 10.1016/j.jneumeth.2007.09.022 18031824

[48] Lemm S, Blankertz B, Curio G, Muller K (2005). Spatio-spectral filters for improving the classification of single trial EEG. IEEE Transactions on Biomedical Engineering.

[49] Ojala, M. & Garriga, G. C. Permutation Tests for Studying Classifier Performance. In *2009 Ninth IEEE International Conference on Data Mining*, 908–913 (IEEE, Miami Beach, FL, USA. http://ieeexplore.ieee.org/document/5360332/ (2009).

[50] Good, P. *Permutation Tests: A Practical Guide to Resampling Methods for Testing Hypotheses* (Springer, 2nd edn (2000).

