# Supplementary material for: Information gain modulates brain activity evoked by reading
Source: Sci Rep. 2020 May 6;10:7671. doi: 10.1038/s41598-020-63828-5 (PMC7203262; doi:10.1038/s41598-020-63828-5)
Supplement: Supplementary file 1 — Supplementary information [file 41598_2020_63828_MOESM1_ESM.pdf]

Supplementary Information for

Information gain modulates brain activity evoked by reading

Lauri Kangassalo, Michiel Spapé, Niklas Ravaja, and Tuukka Ruotsalo

The correspondence should be addressed to Tuukka Ruotsalo: tuukka.ruotsalo@helsinki.fi

This PDF file includes:

Supplementary text
Figs. S1 to S2
Tables S1 to S3
Caption for Movie S1
References for SI reference citations

Other supplementary materials for this manuscript include the following:

Movie S1

Supporting Information Text

Information gain

Model. The information gain (IG) of a word is defined as the change in Shannon entropy over documents $H(D)$ when new evidence w is observed. Given that entropy is a measure of uncertainty, information gain is the decrease in uncertainty concerning documents upon observing evidence. Formally, information gain is defined as $IG(D|w) = H(D) - H(D|w)$. To compute these entropies, we need to define a prior probability distribution for the documents $P(d)$, and a generative probability for a document given a word $P(d|w)$.

$P(d)$ is the probability of a document being drawn from the document pool without any observed words. $P(d)$ could be used to introduce a priori information about the documents, such as document length, popularity, or newness. However, we are particularily interested in how much information gain is achieved when perceiving a word w, independent of any artificially introduced prior information. Thus, we define each document to be equally likely to be drawn from the document pool, formally $P(d) = \frac{1}{|D|}$.

The generative probability of a document given a word, $P(d|w)$, is defined using a derivative of a generative likelihood model (1). First, we assign a probability $P(w|M_d)$ for a word w given a document model M_d for a document d. The document model is a bag-of-words representation of a document, in which the order of the words is disregarded, and only the frequency of each word is preserved. The probability of a word occurring generated by a document can be estimated as:

$$P(w|M_d) = \frac{f_{w,d}}{f_d},$$

such that

$$\sum_{w \in M_d} P(w|M_d) = 1,$$

where $f_{w,d}$ stands for word frequency for word w in document d and f_d is the total amount of words in d.

Next, since we are interested in the distribution of documents given a word, we calculate $P(d|w)$. By utilizing Bayes' rule this becomes:

$$P(d|w) \propto P(w|d)P(d),$$

where $P(t)$ can be ignored, since it is the same for all d. Since we defined that the documents have an uniform prior probability, the equation can be simplified further:

$$P(d|w) \propto P(w|d)$$

Due to this, $P(w|d)$ can be used to compute the probability of a word "generating" a document.

We are now ready to compute the a priori entropy over documents $H(D)$ and the entropy over documents when observing a word $H(D|w)$. By using the definition of entropy and conditional entropy, we get

$$H(D) = -\sum_{d \in D} P(d) \log_2 P(d)$$

and

$$H(D|w) = -\sum_{d \in D} P(d|w) \log_2 P(d|w)$$

Since $P(d)$ is uniform, $H(D)$ will yield the maximum entropy for the given set of documents, formally $H(D) = \log_2(|D|)$. From here it follows that we now have a model for computing the information gain of a word w given a collection of documents D:

$$\begin{aligned} IG(D|w) &= H(D) - H(D|w) \\ &= \log_2(|D|) + \sum_{d \in D} P(d|w) \log_2 P(d|w) \end{aligned}$$

To understand how the measure of information gain works, let us view how the generative distribution of documents changes when conditioned on different words. Consider a collection of 50 Wikipedia articles D'. A language model is generated for each of these documents as specified above, and the generative probabilities $P(d|w)$ are computed for all $d \in D'$ given the words *the*, *small*, and *cat*. These words are examples of low, medium, and high information gain words, respectively. Figure S1 displays the probability distributions of $P(D'|w)$ for each of the aforementioned words, alongside with the conditional entropy of each distribution $H(D'|w)$. We see that $H(D'|w)$ is highest for the word *the*, which is due to the fact that the frequency of *the* is roughly the same in all of the documents. This implies that *the* is not very good at discriminating documents from each other. On the other hand, the word *cat* occurs only in one document in our limited collection. This makes the entropy of the document distribution fall to zero, because there is no uncertainty about a document given the word; we are certain that the document is the one in which *cat* occurs. In a larger collection of documents, say, one consisting millions of documents, it

would be very unlikely for a word to occur in only one document. Lastly, the word *small* falls between the words *the* and *cat* in terms of entropy. It occurs in some documents but not all, and thus is somewhat descriptive in terms of documents. To study the information gains of these three words, we simply subtract the conditional entropy from the a priori entropy, which for our collection is $H(D') = \log_2 50 = 3.91$:

$$IG(D'|the) = 3.91 - 3.71 = 0.20$$
$$IG(D'|small) = 3.91 - 1.53 = 2.38$$
$$IG(D'|cat) = 3.91 - 0.0 = 3.91$$

We see that the highest information gain of these three words is achieved with the word *cat*, with the word *the* having the least information gain, and *small* falling between these two. To conclude, words that occur only in select few documents with varying frequencies will tend to have a higher information gain than those words that occur in great many documents with approximately equal frequency. Thus, information gain is an estimate of the information gained on a topic upon observing a particular word.

.

Computation of information gain. In the present study, information gain of each word was computed from the English Wikipedia using the aforementioned model. Document models of all of Wikipedia's articles were generated. Prior to constructing these models punctuation marks were removed from the text and the words were stemmed using the Porter stemming algorithm (2). The Porter stemmer removes the suffixes of words, attempting to map words with similar meanings to one word. For example, the following words:

`connect, connected, connecting, connection, connections`

all map to the stem `connect` and words

`cat, cats`

both map to the stem `cat`.

A word likelihood model was constructed using the aforementioned models. Using these models, information gain was computed for each of the stemmed words. Words with information gain in the 75th percentile were labelled as high information gain words (label 1), and words with information gain less than the 75th percentile low information gain words (label 0). These labels were employed for data visualisation and classifier training, but not for significance testing, for which continuous values of information gain were used. A histogram of the occurrences of information gain of words can be seen in Figure 1 (left).

Technical details of experimental procedure and data analysis

Apparatus and stimuli. Words were presented with an 18-point Lucida Console black typeface at the centre of the 19" LCD screen. They were shown against a silver (RGB 82%, 82%, 82%) background in the middle of a 300 x 100 pixel pattern mask. The mask was a black rectangle with a grid-like pattern, with an opening to show the word. This was used to control the degree to which word length affected light reaching the eyes (i.e. To make sure longer words were not tantamount to more black pixels on the screen). Sentence separators were word-like character repetitions consisting of 4 to 9 numbers (`3333333`) or other non-alphabetic characters (`&&&&&&`), which were designed to mimic the same early visual activity as words without evoking psycholinguistic processing.

The screen was positioned approximately 60 cm from the participants and was running at a resolution of 1680 x 1050 and a refresh rate of 60 Hz. Stimulus presentation, timing, and EEG synchronization were controlled using E-Prime 2 Professional 2.0.10.353 on a PC running Windows XP SP3. EEG was recorded from 32 Ag/AgCl electrodes, positioned on standardised (using EasyCap elastic caps, EasyCap GmbH, Herrsching, Germany), equidistant electrode sites of the 10 - 20 system via a QuickAmp (BrainProducts GmbH, Gilching, Germany) amplifier running at 2000 Hz. Additionally, the electro-oculogram for vertical eye movements (and eye blinks) and horizontal eye movements was recorded using bipolar electrodes positioned respectively 2 cm superior/inferior to the right pupil and 1 cm lateral to the outer canthi of both eyes.

Formal definitions of the Linear Mixed Models. The significance of the findings was tested with Likelihood Ratio Tests (LRTs) between an alternative hypothesis model and a null hypothesis model. The initial models were designed according to the "keep it maximal" -principle (3). Due to convergence failures, however, we dropped the random effects explaining the least variance and refit the models until convergence was achieved, as suggested in (3, 4).

Formally, the initial models were specified as follows. Alternative hypothesis model:

$$Y_{pi} = (\beta_1 + P_{1p})G_i + (\beta_2 + P_{2p})L_i + (\beta_3 + P_{3p})F_i + (\beta_4 + P_{4p})C_i + \beta_5 Z_{pi} + P_{0i} + I_i + \beta_0 + e_{pi}.$$

Null hypothesis model:

$$Y_{pi} = P_{1p}G_i + (\beta_2 + P_{2p})L_i + (\beta_3 + P_{3p})F_i + (\beta_4 + P_{4p})C_i + \beta_5 Z_{pi} + P_{0i} + I_i + \beta_0 + e_{pi}.$$

Fixed effects in the models were information gain (G_i), word length (L_i), word log-frequency in the whole corpus (F_i), word class (content/functional word) (C_i), and document interest preference (Z_i), for word i. Their corresponding slopes were β_1,

β_2, β_3, β_4, and β_5, respectively. The random intercepts were the participant ($P_{0p} \sim N(0, \tau_0^2)$ for participant p), and the item (word) ($I_i \sim N(0, \gamma^2)$). Additionally, the model had a random by-participant slope for the effects of information gain, word length, word log-frequency, and word class ($P_{1p} \sim N(0, \tau_1^2), P_{2p} \sim N(0, \tau_2^2), P_{3p} \sim N(0, \tau_3^2)$, and $P_{4p} \sim N(0, \tau_4^2)$, respectively. β_0 is the overall intercept and $e_{pi} \sim N(0, \sigma^2)$ represents the general error term. The null model was the same as the alternative hypothesis model, except that the fixed effect of information gain was omitted.

After dropping the effects explaining the least variance to achieve convergence, the alternative hypothesis model was formulated as:

$$Y_{pi} = \beta_1 G_i + \beta_2 L_i + \beta_3 F_i + \beta_4 C_i + \beta_5 Z_{pi} + P_{0i} + I_i + \beta_0 + e_{pi}.$$

The null model was constructed by removing the fixed effect of information gain, as above. This formulation was used to compute the results displayed in Table 1.

Since LMMs without a random slope structure may have an increased Type 1 error rate (3), we wanted to ensure that we achieved similar results from the full (non-converging) and reduced (converging) models. Thus, we compared their performance as seen in Table S1. The table displays the Akaike's Information Criterion (AIC), which measures the tradeoff between the goodness-of-fit and model simplicity (5). AIC depends on the component tested and is sometimes lower (better) on the full model and sometimes on the reduced model. Thus, we find that the evidence is not fully conclusive as to which model (full or reduced) provides a better fit for the data. Furthermore, the table displays the χ^2 values of LRT tests between alternate (effect of information gain included) and null (effect of information gain omitted) hypothesis models. The χ^2 values are mostly similar, with the exception of the P300 component, which has a much lower χ^2 value in the reduced model. We can conclude that the results do not change substantially due to the use of a model without random slopes.

Component		Full	Reduced
EPS	AIC	155017	155023
	χ^2	5.29	5.98
P200	AIC	151894	151889
	χ^2	4.39	4.68
P300	AIC	159092	159111
	χ^2	7.63	2.72
N400	AIC	160943	160980
	χ^2	7.37	7.73
P600	AIC	160170	160159
	χ^2	3.43	3.46

Table S1. Akaike's information Criterion and χ^2s of null vs. alternative model for the full model (no convergence) and reduced model (convergence). The χ^2 values in the right column match with the results reported in Table 1.

Information gain prediction

Classifier details and feature engineering. Since we wanted the classifier to utilize both the spatial attributes (channels) as well as the temporal attributes (time w.r.t. stimulus onset) of the data, all channels and sufficient temporal resolution was used to determine classifier features. The tensor $X^{m \times c \times t}$ represents the preprocessed EEG recording for each participant, with m cleaned epochs, c channels and t time points. To reduce the dimensionality of the data, the time points were divided to $t' = 8$ equidistant windows between 0ms and 1000ms, and the average voltage of each of these windows was computed, resulting in a $X^{m \times c \times t'}$ tensor. This led to time windows spanning 80ms. Furthermore, the channels and time windows were concatenated together, resulting in a $X^{m \times c \cdot t'}$ spatio-temporal feature matrix. Essentially, the classifier was trained with all of the available data, and the feature engineering decisions were not informed by the statistical significance performed on the ERPs. This feature engineering procedure follows standards for single-trial ERP classification (6). Since the data is of a relatively high dimensionality ($32 \cdot 8 = 256$) compared to the number of data points (approximately 1400 per training set), LDA with shrinkage was employed. The tuning parameter for shrinkage was chosen with the Ledoit-Wolf -lemma (7).

To be able to evaluate the classifiers, the epochs of each participant were split to eight blocks $B = \{b_0, ..., b_7\}$ coinciding with the eight reading tasks in the EEG measurement experiment. Consequently, each block consisted of the epochs for two documents. A classifier was trained for each block b_i so that each of these classifiers used seven of the other available blocks as a training set $X_{\{B \setminus b_i\}}^{(m-m_i) \times c \cdot t'}$, and were evaluated on the test set $X_{b_i}^{m_i \times c \cdot t'}$.

The classifiers were trained with the information gain labels (low/high). The split at the 75th percentile resulted in imbalanced classes; however, LDA has been shown to be robust against class imbalances (8, 9).

Classifier performance evaluation. The performance of the classifier was measured with the Area under the ROC curve (AUC). This measure was chosen because AUC combines the true positive and false positive rate, and thus gives sufficient performance estimates when the classes are imbalanced. In the case of imbalanced classes, the classifier will tend to predict the dominant class (in this case the high IG class), which causes a standard accuracy measure to give overconfident estimates of performance.

The classifier performance was evaluated with permutation tests. The classifier was trained with permuted class labels to reveal if the classifier had learnt any real class structure in the data. With a sufficiently high number of permutations this

produces permutation-based p-values (10). The null hypothesis is that the class labels and brain activity are independent of each other. A small p-value indicates that the classifier is able to find some meaningful structure of the brain activity that correlates with the class labels (high/low information gain). We ran $k = 1000$ permutations for each subject, so k classifiers with randomly permuted labels were trained for each subject, and their AUCs were compared to the AUC of the actual classifier to produce the p-values. To obtain the AUCs for each subject, we calculated the mean of the AUCs of the per-block classifiers.

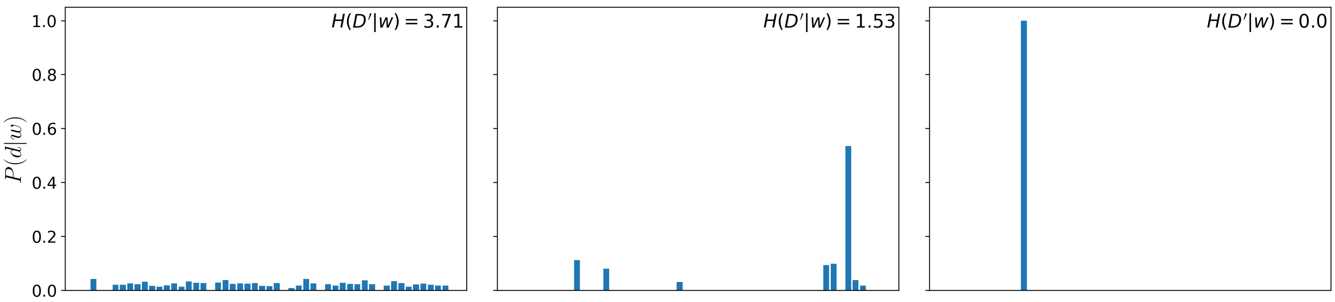

Fig. S1. Probability distributions over 50 randomly chosen Wikipedia documents for the words 'the', 'small' and 'cat'. Conditional entropies ($H(D'|w)$) of the distributions are shown in the upper right corner of each plot.

Table S2. EEG preprocessing details.

Subject	Threshold (μV)	Trials recorded	Trials dropped	Channels dropped
S01	57,42	1 941	388	None
S02	33,88	1 961	392	Fp1, Fp2, TP9, TP10, FT10
S03	65,54	1 936	387	Fp1, Fp2
S04	30,64	1 986	397	Fp1, Fp2, P7
S05	31,19	1 959	391	Fp1, Fp2, F7, TP9, TP10
S06	51,04	1 960	392	Fp1, Fp2, O2
S07	27,98	1 869	373	TP10
S08	62,90	1 958	391	Fp1, Fp2, TP9
S09	47,25	1 818	363	None
S10	28,69	2 026	405	Fp1, Fp2, O2
S11	57,04	1 939	387	None
S12	40,61	1 944	388	Fp1, Fp2, F7, TP9
S13	35,28	1 869	379	Fp1, Fp2
S14	29,96	1 981	396	Fp1, Fp2, F7, FT9, FT10
S15	44,96	1 906	381	Fp1, Fp2, F7

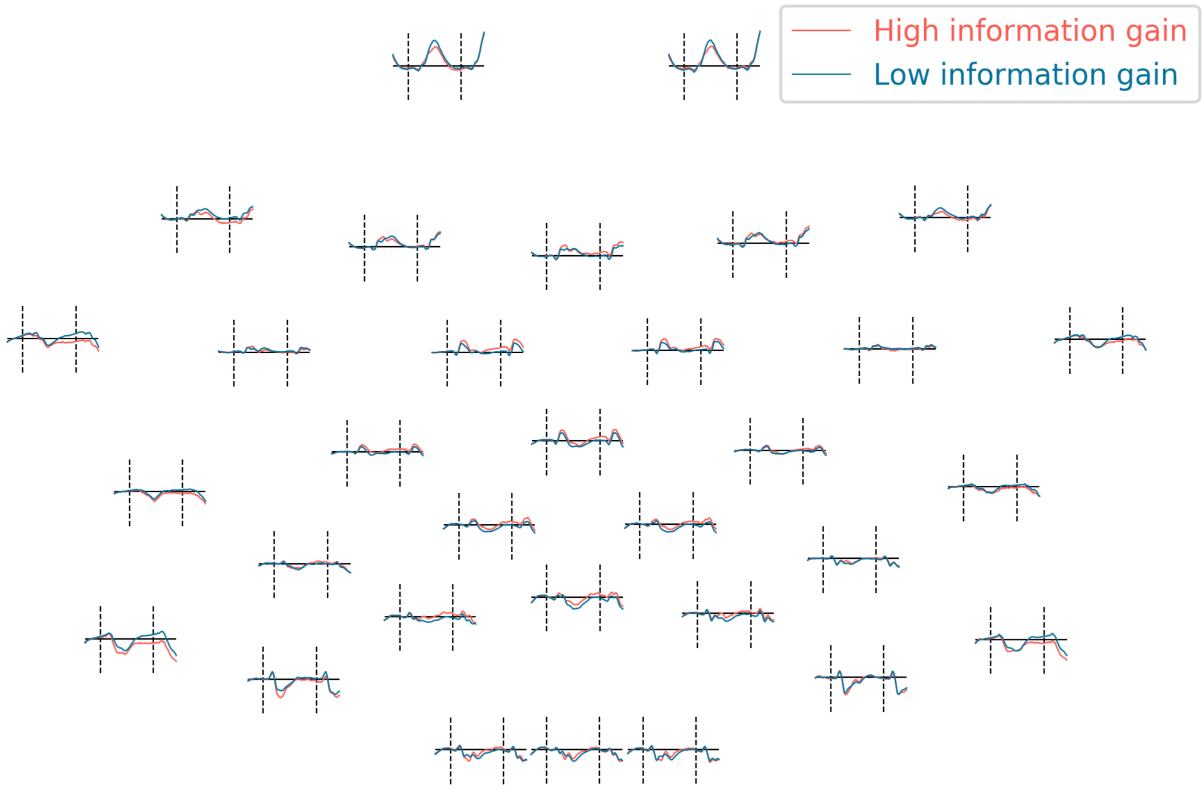

Fig. S2. ERPs for high and low information gain words for all channels. Dashed lines mark stimuli onsets. The averages for channels Fp1 and Fp2 are dominated by the measurements of only a few participants, as the said channels were interpolated on most of the participants.

Table S3. Top 5 words per topic sorted by classifier confidence (predicted) for class membership (high/low information gain) and by true class membership (high/low information gain). All words are converted to lower case.

	Top/bottom 5 words in information gain class:			
Document topic	High IG Predicted	High IG True	Low IG Predicted	Low IG True
atom	quantum	neutrons	or	the
	successfully	isotope	have	and
	microscope	protons	such	a
	positively	radioactive	one	of
	only	nucleus	that	is
automobile	regarded	motorcar	one	the
	affordable	benz	many	and
	million	baggage	after	in
	automobile	electrified	soon	a
	billion	risen	or	of
bank	deficits	berenberg	either	the
	surpluses	paschi	is	and
	regulated	institutionalised	on	in
	liabilities	surpluses	are	a
	highly	siena	existing	of
bicycle	automobiles	sprockets	around	the
	bicycles	bicyclist	century	and
	worldwide	pneumatic	to	in
	played	cyclist	an	a
	changed	upright	first	of
bill clinton	arkansas	boomer	who	the
	democrat	42nd	an	and
	born	peacetime	over	in
	described	arkansas	to	a
	agreement	jefferson	in	of
brain	generating	synapses	as	the
	invertebrate	cortex	typical	and
	special	sensory	center	in
	hormones	cerebral	a	a
	control	hormones	with	of
cat	killing	housecat	with	the
	housecat	felids	for	and
	mammal	purring	as	in
	indoor	mewing	such	a
	despite	felines	being	of
communism	marxism	marxism	has	the
	maximized	socioeconomic	in	and
	distinction	marx	and	in
	socialized	dictatorship	absence	a
	marx	recycling	is	of
euro	dollar	eurozone	has	the
	eurozone	banknotes	into	and
	december	currency	as	in
	following	euro	july	of
	european	coins	2002	was
football	opposing	torso	as	the
	penalty	spherical	are	and
	rectangular	codified	into	in
	eleven	outfield	were	a
	touch	goalkeepers	to	of
india	independence	pluralistic	to	the
	asia	indus	nation	and
	independent	multilingual	in	in
	civilisation	mahatma	vast	a
	mahatma	civilisation	of	of

learning	machines	habituation	human	the
	consciously	factual	to	and
	reinforcing	conscious	activities	in
	habituation	synthesizing	of	a
	intelligent	consciously	and	of
machine learning	filtering	subfield	by	the
	algorithm	unsupervised	with	and
	unsupervised	spam	include	in
	outputs	conflated	search	a
	deals	filtering	that	of
michael jackson	professional	moonwalk	an	the
	philanthropist	philanthropist	to	and
	publicized	robot	such	in
	1982	thriller	as	a
	brothers	dancer	with	of
money	medium	banknotes	to	the
	repayment	fiat	and	and
	banknotes	intrinsic	its	in
	intrinsic	deferred	accepted	a
	market	repayment	of	of
ocean	hydrosphere	hadean	in	the
	impetus	hydrosphere	and	and
	emergence	oceanographers	on	in
	divisions	saline	which	a
	contains	impetus	an	of
painting	spiritual	airbrushes	to	the
	craftsmen	sponges	act	and
	surface	knives	or	in
	brush	craftsmen	be	a
	outside	pigment	such	of
plato	philosophical	socrates	is	the
	aristotle	socratic	been	and
	academy	plato	in	in
	athens	platonism	perspective	a
	higher	aristotle	have	of
politics	practice	adversaries	in	the
	employed	sovereign	or	and
	international	discourse	which	in
	influencing	civic	wide	a
	institutions	warfare	among	of
rome	michelangelo	bramante	to	the
	bramante	bernini	chapel	and
	province	sistine	for	in
	baroque	tiber	in	a
	architecture	michelangelo	was	of
savanna	unbroken	unbroken	also	the
	hemisphere	herbaceous	of	and
	grassland	savannas	and	in
	majority	savanna	common	a
	seasonal	savannah	by	of
schizophrenia	syndromes	contributory	a	the
	characterized	antipsychotic	have	and
	schizophrenia	dopamine	often	a
	unclear	auditory	number	of
	important	schizophrenia	receptor	is
school	teenagers	homeschooling	a	the
	homeschooling	compulsory	but	and
	building	vocational	have	in
	an	seminary	the	a
	dedicated	teenagers	who	of

society	institutions<br>ant<br>insofar<br>otherwise<br>societies	criminology<br>subculture<br>interpersonal<br>insofar<br>ant	on<br>used<br>and<br>by<br>that	the<br>and<br>in<br>a<br>of
star	gaseous<br>primarily<br>gravity<br>plasma<br>source	asterisms<br>luminous<br>nebula<br>helium<br>gaseous	to<br>collapse<br>a<br>the<br>space	the<br>and<br>a<br>of<br>is
telephone	transmissions<br>telecommunications<br>landline<br>microphone<br>numeric	earphone<br>keypad<br>landline<br>microphone<br>cellular	on<br>two<br>by<br>such<br>first	the<br>and<br>in<br>a<br>of
time	astronomy<br>occupied<br>debate<br>quantities<br>durations	technologists<br>judgement<br>temporal<br>astronomy<br>sensation	from<br>in<br>component<br>was<br>as	the<br>and<br>in<br>a<br>of
volcano	eruption<br>temperature<br>tectonic<br>surface<br>atmosphere	troposphere<br>droplets<br>magma<br>plumes<br>crust	to<br>can<br>is<br>lower<br>on	the<br>and<br>in<br>a<br>of
wife	varies<br>cultures<br>heterosexual<br>separated<br>widow	heterosexual<br>marital<br>spouse<br>obligations<br>widow	from<br>of<br>also<br>may<br>in	the<br>and<br>in<br>a<br>of
wine	chemical<br>thousands<br>egyptians<br>appearance<br>nutrients	6000bc<br>yeasts<br>ferment<br>fermented<br>beverage	so<br>lets<br>is<br>has<br>and	the<br>and<br>in<br>a<br>of

Movie S1. Animation of differential scalp topographies for low/high information gain words for the time interval 0 - 1000 ms post-stimuli. The topographies advance in steps of 5 ms.

References

1. Manning CD, Raghavan P, Schütze H (2008) Language models for information retrieval in *Introduction to information retrieval*. (Cambridge University Press, New York). OCLC: ocn190786122.
2. Porter M (1980) An algorithm for suffix stripping. *Program* 14(3):130–137.
3. Barr DJ, Levy R, Scheepers C, Tily HJ (2013) Random effects structure for confirmatory hypothesis testing: Keep it maximal. *Journal of Memory and Language* 68(3):255–278.
4. Matuschek H, Kliegl R, Vasishth S, Baayen H, Bates D (2017) Balancing type i error and power in linear mixed models. *Journal of Memory and Language* 94:305 – 315.
5. Akaike H (1998) Information theory and an extension of the maximum likelihood principle in *Selected papers of hirotugu akaike*. (Springer), pp. 199–213.
6. Blankertz B, Lemm S, Treder M, Haufe S, Müller KR (2011) Single-trial analysis and classification of ERP components — A tutorial. *NeuroImage* 56(2):814–825.
7. Ledoit O, Wolf M (2004) A well-conditioned estimator for large-dimensional covariance matrices. *Journal of Multivariate Analysis* 88(2):365–411.
8. Xue JH, Titterington DM (2008) Do unbalanced data have a negative effect on LDA? *Pattern Recognition* 41(5):1558–1571.
9. Xue J, Hall P (2015) Why Does Rebalancing Class-Unbalanced Data Improve AUC for Linear Discriminant Analysis? *IEEE Transactions on Pattern Analysis and Machine Intelligence* 37(5):1109–1112.
10. Ojala M, Garriga GC (2009) Permutation Tests for Studying Classifier Performance in *2009 Ninth IEEE International Conference on Data Mining*. (IEEE, Miami Beach, FL, USA), pp. 908–913.
